# Development of an Educational Game to Set Up Surgical Instruments on the Mayo Stand or Back Table: Applied Research in Production Technology

**DOI:** 10.2196/games.6048

**Published:** 2017-01-10

**Authors:** Crislaine Pires Padilha Paim, Silvia Goldmeier

**Affiliations:** ^1^ Institute of Cardiology of Rio Grande do Sul University Foundation of Cardiology Porto Alegre Brazil

**Keywords:** nursing education research, educational technology, perioperative nursing

## Abstract

**Background:**

Existing research suggests that digital games can be used effectively for educational purposes at any level of training. Perioperative nursing educators can use games to complement curricula, in guidance and staff development programs, to foster team collaboration, and to give support to critical thinking in nursing practice because it is a complex environment.

**Objective:**

To describe the process of developing an educational game to set up surgical instruments on the Mayo stand or back table as a resource to assist the instructor in surgical instrumentation training for students and nursing health professionals in continued education.

**Methods:**

The study was characterized by applied research in production technology. It included the phases of analysis and design, development, and evaluation. The objectives of the educational game were developed through Bloom’s taxonomy. Parallel to the physical development of the educational game, a proposed model for the use of digital elements in educational game activities was applied to develop the game content.

**Results:**

The development of the game called “Playing with Tweezers” was carried out in 3 phases and was evaluated by 15 participants, comprising students and professional experts in various areas of knowledge such as nursing, information technology, and education. An environment was created with an initial screen, menu buttons containing the rules of the game, and virtual tour modes for learning and assessment.

**Conclusions:**

The “digital” nursing student needs engagement, stimulation, reality, and entertainment, not just readings. “Playing with Tweezers” is an example of educational gaming as an innovative teaching strategy in nursing that encourages the strategy of involving the use of educational games to support theoretical or practical classroom teaching. Thus, the teacher does not work with only 1 type of teaching methodology, but with a combination of different methodologies. In addition, we cannot forget that skill training in an educational game does not replace curricular practice, but helps.

## Introduction

Education, in an enlarged view, covers the formative processes that occur at work in school learning and human coexistence. Current research suggests that digital games can be used effectively for educational purposes at any level of training [1,2].

In 2015, the Horizon Report identified game-based learning as an area likely to have major impact on learning in the next 2 years. The discovery of this report is indicative of the growing interest in the use of digital games technology with effective projection in education [3].

An educational game is defined as an instructional method, which requires that the learner participates in a competitive activity with preset rules [4]. With educational games, learners have an opportunity to experiment with decision-making and problem-solving in a risk-free active learning environment [5]. Therefore, educational games are considered experiential learning methods that may contribute positively to students’ learning.

Generally, educators use active learning approaches to keep up with new interesting data, promote information retention, and stimulate critical thinking. This is especially important when the subject is complex or tedious [6].

Surgical centers are considered high risk scenarios extremely susceptible to errors dominated by pressure and stress [7,8]_._ It is well known that students from different fields of medical science are exposed to several stress factors throughout clinical education processes inside the operating room [9-11]. The level of anxiety can increase when some factors are associated [12], such as interpersonal communication, humiliating experiences, educational environment, clinical experiences, and unpleasant emotions [10].

Perioperative nursing has a serious agenda with significant content that can be adapted to the game environment for teaching, improving proficiency and self-development, and conducting outcomes of research for best practices and improvement initiatives. The prospect of using serious games creates an exciting—and maybe for some, a frightening—future for those who face this oncoming technology [13].

Perioperative nursing educators can use games to complement curricula in guidance and staff development programs, foster team collaboration, and give support to critical thinking in nursing practice because it is a complex environment [6].

If a serious game could help nurses overcome fear of new procedures, learn changes in the sequencing of events, reduce the stress of a situation, or help prevent errors, then the patient will be the ultimate winner [13].

In this context, the search for quality education of health professionals and their services is based on guidelines. As an example, we mention the Safe Surgery Saves Lives campaign, whose main objective is to improve the safety of surgical care around the world by defining a core set of safety standards that can be applied in all countries and settings. The protocol includes the prevention of infection in surgical sites, safe anesthesia, safe surgical teams, and surgical care indicators [14]. The idea is that the educational game can help in this search process for excellence in teaching in the care of surgical patients.

An example in international literature in the surgical nursing area is the QuizBowl game that was presented at the Annual Congress of the Association of Perioperative Registered Nurses 2003 to 2010 [6]. Moreover, in the general nursing area, a Jeopardy-style game called “Nursopardy” is used to reinforce the fundamentals of nursing material, aiding in students’ preparation for a standardized final exam [15]. Still under development is an in-home and community care settings game [16]. The e-Baby Serious Game was built to feature the simulated environment of an incubator, in which the user performs a clinical evaluation of the respiratory process in a virtual preterm infant [17]. LISSA is another serious game that considers all the steps of the cardiopulmonary resuscitation (CPR) flowchart [18].

Thus, the aim of the study was to describe the process of developing an educational game to set up surgical instruments on the Mayo stand and back table as a resource to assist the instructor in training nursing students and health professionals in continued education.

## Methods

### Study Design

The study was based on applied research in technology production [19], which was adopted because it dealt with the process of developing or creating a new product, activity or service, namely, a learning management system in nursing where the theme was to develop an educational game.

This study was approved by the Research and Ethics Committee of the Institute of Cardiology of Rio Grande do Sul state.

The Galvis Panqueva methodology was used to create an educational game [20] because of its clarity and cohesion, making the process more objective. This methodology comprised 3 phases: analysis and design, development, and evaluation.

A constructivist learning theory was employed to achieve the objectives of the educational game in development. This requires that learners use various tools to access content information, derive meaning, and develop knowledge of how to work through a scenario and solve given problems. Guiding this construction of knowledge, Bloom’s taxonomy offers 6 levels of competencies to acquire knowledge about a topic moving from simple to complex levels: knowledge, comprehension, application, analysis, synthesis, and evaluation [21].

Parallel to the physical development of the educational game, a proposed model for the use of digital elements for educational game activities was used for the development of game content [22].

### The Phases

#### Phase 1: Analysis and Design

The target audience, choice of subject, educational goals, outlining of the content, and technological infrastructure were defined in the analysis phase.

The target audience chosen was surgical instrumentation students and nursing health professionals in continued education.

The choice of subject was the setup of surgical instruments on the Mayo stand and the back table with basic instruments.

Thus, the educational goals for the cognitive domain were established by considering that at the end of the game, the student was able to set up surgical instruments on the table and understand the organizational sequence when applying it to the setup of the Mayo stand and back table. Additionally, the student would be able to analyze organization of the operating table, synthesize organization of the operating table in accordance with the procedure and types of instruments needed, and finally evaluate the acquisition of knowledge with increasing score.

In order to approach the subject, the content of basic types of surgical instruments and surgical time were considered important, and for the technological infrastructure, the support of a team of game designers.

In the game design phase, the proposal was to create an environment with instruments on a basic tray used in general surgery, for example, hernioplasty and postectomy, in which the student had to arrange them according to the model.

The company hired to produce the game planned the navigation structure, navigation map design, learning environment, and the interface design. As for the system tools, the need for help buttons to facilitate solving doubts was stipulated.

#### Phase 2: Development

The development stage in the construction consisted of an educational game developed by the researchers and 3 game designers. Its construction comprised the months of April 2015 to February 2016.

According to the proposed model for the use of gamification elements, we cite elements pertaining to this game in the following points:

Mission: To set surgical instruments on the Mayo stand or back table according to the model in virtual tour mode while considering the 6 surgical times: cutting, grasping, hemostatic, retractors, special, and suture.

Plot: In the game, the player assumes the role of a surgical nurse in the operating room. In the learning mode, the player has no specific time to finalize the setup table, but in assessment mode is given 7 minutes, with a basic instrumental tray used in general surgery (hernioplasty and postectomy). The player has to recreate a model setup of surgical instruments proposed in the virtual tour.

Character: A character called Metzenbaum was created to represent a surgical nurse.

Challenges: Increase the score with each attempt, until one reaches the appropriate level of 70% of correct positions. After placing the instruments in each step, the player has to click the correct button, which results in the following warnings: “Try again,” “Congratulations, you have reached 70% or more,” “Great you hit 100%.”

Specific objectives: Place the instruments in the location set for each surgical time.

Resources, collaboration, or help: In the virtual tour mode, the player can view the setup of the surgical table, which includes scientific and popular names and classes of instruments. In learning mode, the player can use the “hint” as many times as needed, showing the location where the instruments should be placed. In either way, the student assessment will not provide any help.

Items or bonus: For each surgical time that is completed with the instruments of the game, the player is awarded a star as a bonus; 6 stars being the maximum.

The game was developed in HTML 5 using the an computer program, which can be used on Windows, Linux, and Mac platform. The instrumental images were photos from the researcher’s personnel file and modeled in *blendere* changed in *gimp*. The game designers built the image of the avatar in surgical nursing.

The game will be hosted on the financial institution's website and a password will be provided to the user.

#### Phase 3: Evaluation

During the stage of the evaluation, students from the surgical instrumentation course and expert professionals in various fields of knowledge related to nursing, information technology, and education were invited to evaluate the learning environment. The evaluators comprised a nonprobabilistic convenience sample for which selection was performed using nonrandom, intentional methods [19].

A specific form based on 3 thematic areas according to the Ergolist Project was used for data collection, distinct for the different areas of professionals [23], using a Likert scale, in which the user had to mark one of the choices: “totally disagree,” “partially disagree,” “indifferent,” “partially agree,” and “totally agree.”

Health education and nursing experts, along with the students evaluated the following thematic areas: (1) *Educational aspects* (if the central theme is significant, if there is coherence of objectives, if theoretical directions are understandable, if exercises and activities are easy to understand, if valuation method is applicable, if autonomy is provided to the user, and if didactic resources helped in solving problems); (2) *Environment interface* (if navigability, accessibility, environment design, and didactic resources are easy to view); and (3) *Interactivity of the system* (if it is easy to use and navigate on the screen).

However, the specific form for information technology professionals contemplated *Response time* (referring to navigability, accessibility, and system feedback to the user);  *Interface quality* (design, colors, menu, and buttons); and  *Tools and resources* (form, presentation, and working operating system).

## Results

Delivery of the first release of the game was in version 1.0 where there were discussions on some topics regarding the layout and background color, the slogan of the institution, use of visual and auditory feedback, and background music. This release featured the home screen, which allowed visualization of the game design with the slogan of the funding institution and a menu with 4 topics: virtual tour, learning, assessment, and credits. The topic of learning highlighted the surgical instruments of a Mayo stand at the center and a scroll bar system on the underside with the instrumentals.

On delivery of the second release in version 1.1, we included the virtual tour screen with images of the instruments, the learning screen with the scroll bar system, instruments, and signalization of hits and fails on the screen. We required adjustments in relation to the position of the instruments (closed and front position) and defined the pilot project (1st testing) with surgical instrumentation students.

The pilot group consisted of 6 surgical instrumentation students. We identified that the sample agreed 100% (6/6) totally or partially with the educational aspects. Therefore, we concluded that the central theme is significant, there is consistency of objectives, the theoretical directions are understandable, the evaluation method is applicable, exercises and activities are easy to understand, it offers autonomy and educational support, and assists in resolution. With regard to the interface, most of the sample totally or partially disagreed as follows: 83% (5/6) compared with navigability and 67% (4/6) with accessibility and design. Regarding the interactivity of the system, 50% (3/6) of the sample agreed that the game screen was easy to navigate and easy to use, but the other half disagreed with this view. The suggestions given were in improving the system, use of sound to signal the hit and fail, time to score, streamlined speed of response commands, to highlight the display of instruments, and increase the amount of tweezers.

After the study, the requests were addressed, such as sound differentiating on hits and fails, positioning and addition of instruments, time recorded on the learning screen, the sum of the points on the evaluation screen, use of 3D image, and buttons to move the instrumental.

The third release in version 1.2 was tested with surgical instrumentation students and professional experts from various fields of knowledge related to nursing, information technology, and education. There were 15 participants, which included 8 surgical instrumentation students, 4 professionals with experience in surgical instrumentation, a professor from the surgical instrumentation course, and 2 IT professionals.

We identified that the entire sample of health professionals and students agreed totally or partially with the educational aspects, showing no difference to the pilot group. With regard to the interface, there was improved acceptance with 77% (10/13) that agreed totally or partially to navigability, 85% (11/13) to accessibility, and 77% (10/13) to design. Regarding system interactivity, 100% of the sample agreed that the game screen is easy to navigate and use.

The suggestions given were to start the game with a total score and subtract points as mistakes were made. Others included: to use a stop button, increase the range of adjustment, improve the viewing tip of the instruments, provide a score below the scroll bar, use as criterion the calculation of points for accuracy and time, delete the fail sound, improve color of the screen with an updated design, adjust the resolution of the instruments, divide the table and highlight the phases, and enable musical background sound.

The IT group rated the response time, interface quality, tools, and resources. Responses showed no particularity, emphasizing that the environment was appropriate for a teaching proposal.

Use of the Galvis Panqueva methodology, in association with educational objectives and digital gaming elements, created for the educational game “Playing with Tweezers,” a virtual environment that teaches instrumentalization in an active participatory fun way. The game offers a learning and evaluation platform facilitating the teaching-learning process.

**Figure 1:**
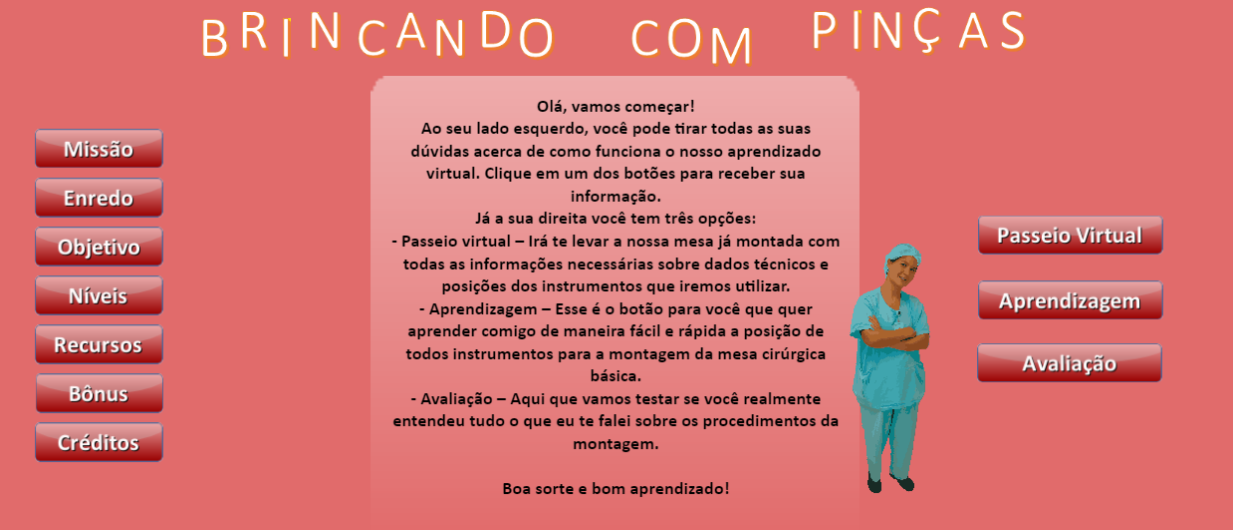
Initial screen.

**Figure 2:**
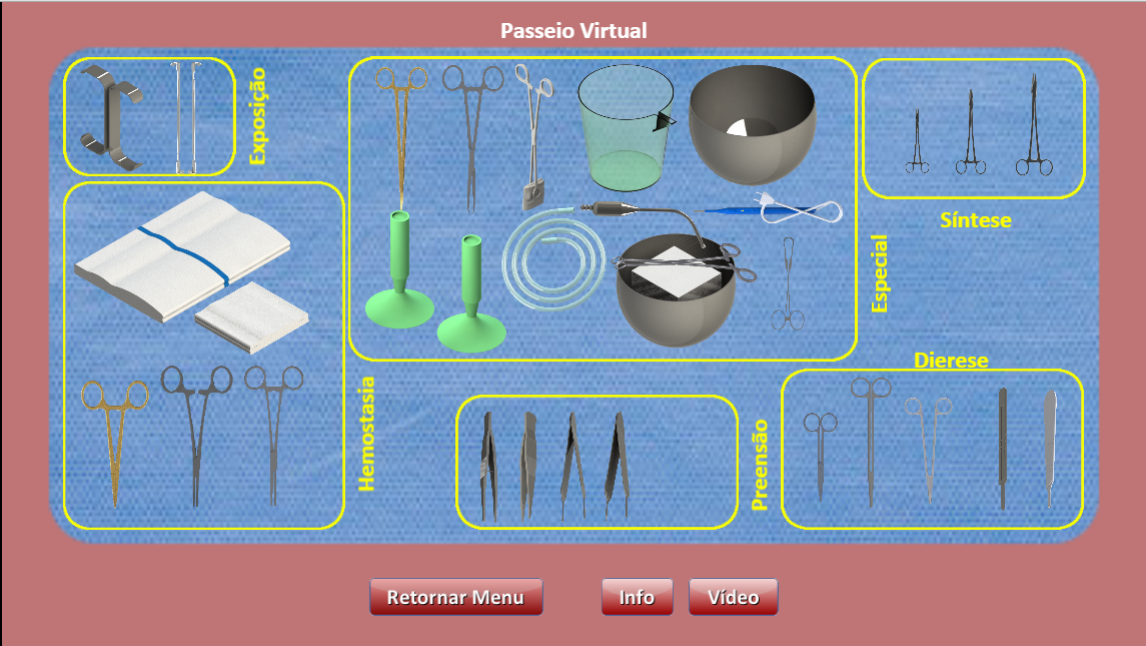
Virtual tour mode for learning.

**Figure 3:**
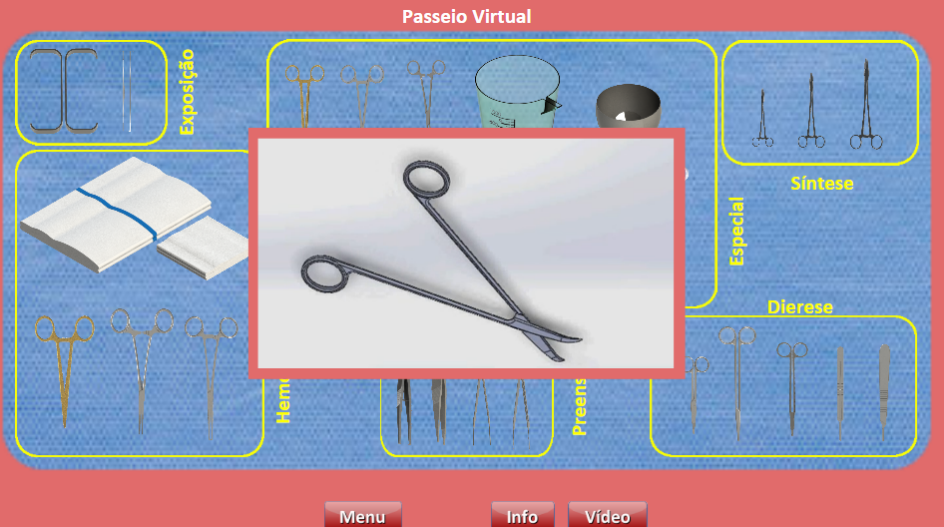
Instrumental in 3D.

**Figure 4:**
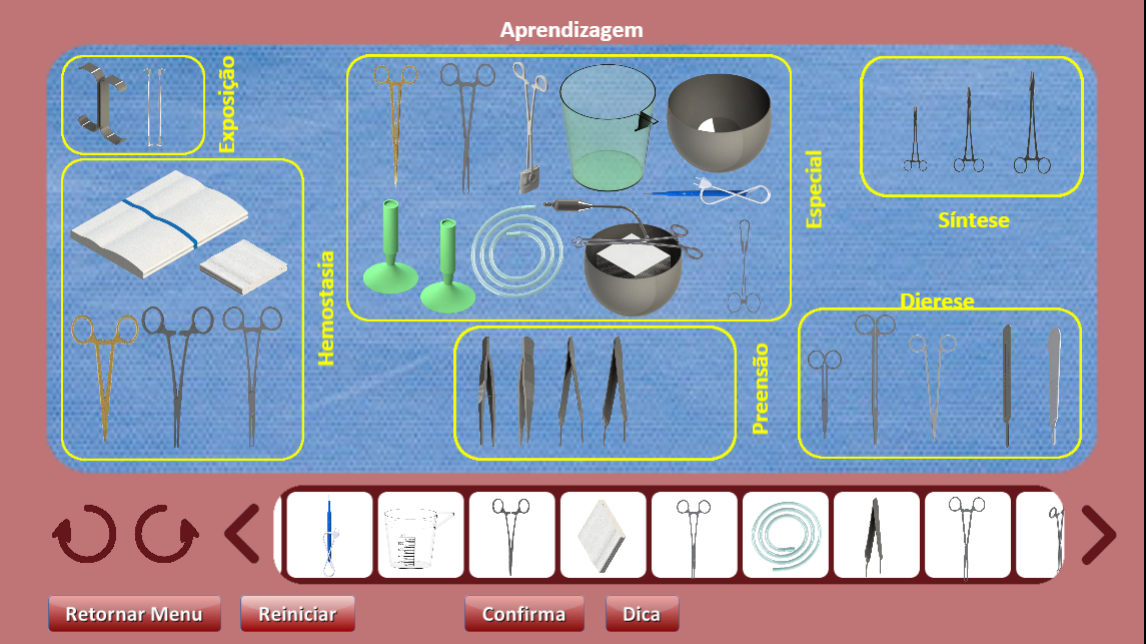
Virtual tour mode for assessment.

**Figure 5:**
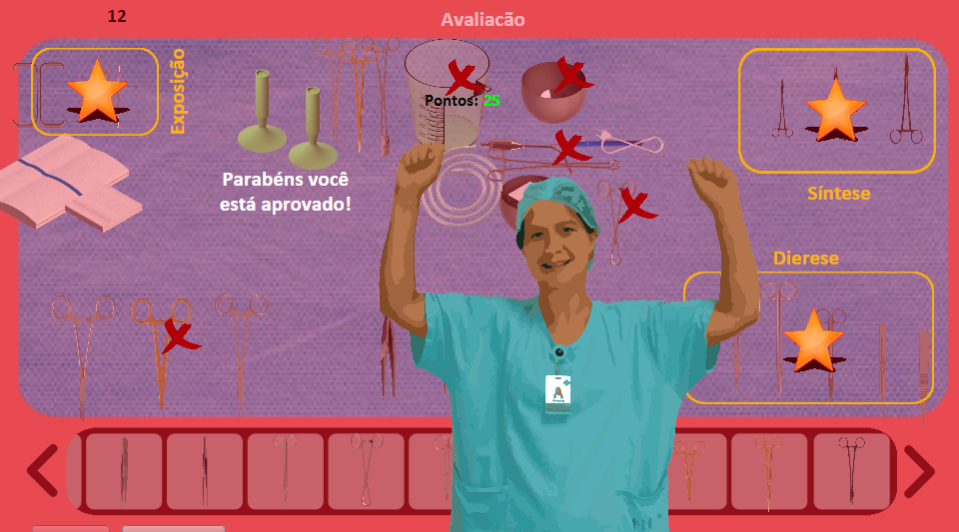
Final screen of the game.

An environment with an initial screen containing menu buttons with rules of the game and the virtual tour modes, learning, and assessment was created. We used a character feature (avatar) to represent the surgical nurses. At the end of the game, if the score was above 70%, a buzzer goes off and the avatar of the surgical nurse signals, raising its hands with a happy expression. In the situation of being below a 70% score, you hear a new sound, and the avatar with hands on its face, obscures the appearance of sadly crying, highlighting the need for greater attention. The screens of the final version of the game are shown in Figures 1-5.

## Discussion

### Principal Findings

The literature on the use of educational games has repeatedly shown that they have a positive effect on leaners [2,6,15,24-27]. Although this study only describes and assesses an educational game to set up surgical instruments on the Mayo stand and back table, the evaluation data of the study suggests there is some level of interest from those within the study population toward the use of a digital game to teach perioperative nursing. This preliminary finding appears to support the existing research in the area and has the potential to add data to an area of research that is underexplored.

Perioperative nursing consists in meaningful content that can be tailored to the targeted teaching game environment, improving proficiency and self-development, and the results that conduct research for best practices and improvement initiatives.

Changes in our health care system are demanding that nurses have to develop new skills and competencies. Having the opportunity to “test-out” various clinical reasoning pathways allows the practitioner to become more aware of how reason is reached and recognize the consequences of this reasoning in action [16].

In the context of medical education, there has been significant research interest in the use of digital games in clinical training, such as for surgical skills training [24]. There is also a small amount of research in the use of digital games for continued education, including studies in their usability for retraining of resuscitation skills [18,25].

For example, an article evaluated the use of LISSA, a serious game that considers all the steps of the CPR flowchart. We analyzed the effect of using LISSA after the introduction of theory and before laboratory practice. The results obtained showed that students who practiced with LISSA performed better in the laboratory sessions than students that only read theoretical material. Therefore, from the results we can conclude that the use of LISSA improves students’ knowledge and skills on CPR. In addition, students feel that LISSA helps them to learn [18].

Another study created a 3D serious game for scenario-based retraining and proved to be effective in advanced life support and supported retention of acquired knowledge and skills in 3 months. The serious game, called EMSAVE, also positively engaged and motivated participants [25].

In the perioperative nursing area, 1 study describes the creation, implementation, and evaluation of a game called “Nursopardy.” Nursopardy was used with first semester students in nursing classes to prepare them for the final exams. The game consisted of 5 categories with a total of 26 questions, involving care management issues, risk reduction, safety and infection control, physiological adaptation, and basic care. Research involved a small sample of 39 students, but concluded that the game is a useful teaching strategy and the students had positive evaluations. The combination of visual, sound, teamwork, and competition worked well to increase the teaching-learning process [15]. This educational game works with the objective to improve critical thinking and decision-making, and proves that there is still a gap in the literature in relation to developing skills in perioperative nursing.

The data obtained in the evaluation of students, specialists in nursing and computer areas, were extremely rewarding and fully validated the objectives of this study. “Playing with Tweezers” proved to be an intuitive virtual environment, visually pleasant, with good navigability and accessibility. It has virtual drive modes, learning, and assessment that facilitate the teaching-learning process.

### Limitations

Some limitations of the study were observed in relation to the handling of this new technology as well as the use of information technology in a small sample size.

Our future work will be centered on the design of new scenarios and the introduction of new characters into the game and will assess the effect of educational gaming for the improvement of knowledge and skills.

### Comparison With Prior Work

There are numerous examples of educational games that can be used in health care specialties, including jeopardy style, concentration, quiz bowls, puzzle formats, crossword puzzles, card games, board games, electronic games, slideshows, and multimedia formats. Regardless of the type of game used, it is important to carry out an assessment of knowledge consolidation [1,5]. There is limited information about the impact of these strategies on learner engagement and outcomes [26]. Further research in this area would be of benefit to both learners and educators alike [1].

A Cochrane review evaluated the effect of educational games on health professionals’ performance, knowledge, skills, attitude, and satisfaction as well as on patient outcomes. Just 2 randomized controlled trials, in which the effect knowledge was not statistically different between the 2 groups, were included. The findings of the systematic review did not confirm nor refute the use of games as a teaching strategy for health professionals. More and better evidence is needed to make practice recommendations. However, those designing and implementing educational games should carefully consider their advantages and disadvantages [5].

A randomized controlled trial was conducted with 145 medical students to compare the effectiveness on the learning outcome of a game-based e-learning instruction with conventional script-based instruction in the teaching of phase contrast microscopy urinalysis under routine training conditions of undergraduate medical students. Of the total, 82 subjects where allocated for training with an educational adventure-game and 69 subjects for conventional training with a written script-based approach (script group). The students in the game group achieved significantly better results in the cognitive knowledge test than the ones in the script group. The mean score was 28.6 for the game group and 26.0 for the script group. Attitudes toward the recent learning experience were significantly more positive with the game group. Students reported having more fun while learning with the game compared with the script-based approach [2].

The gaming platform “They Know” is a strategy game based on team participation designed for use in a variety of educational curricula. In the context of this study, the platform will be used to develop a game for medical students to study anatomy and histology. The goal is to work in cooperation with teammates in order to take control of a home base opposing team across the map. To cross the map, players must answer multiple-choice questions on each node they pass, related to its specific category of learning. During the game, players will be observed by a study coordinator and will be filmed for the research team to review how the players interact with their teammates. The research team will also be working with the study participants to explore the ways in which their knowledge was structured as a result of playing the educational video game [1].

Nursing faculty at a mid-Atlantic historical black college and university introduced “serious gaming” technology to a community health nursing course by using 2 Web-based game simulations (1) Outbreak at WatersEdge: A public health discovery game, and (2) EnviroRisk. This innovation proved to be effective in reinforcing learning and improving student learning outcomes [27].

It is necessary to determine whether learning is taking place and what are the game elements that support learning. More studies are suggested to observe how active-learning strategies, such as games, affect learning outcomes, including exam scores for nursing students.

### Conclusions

The “digital” nursing student needs engagement, stimulation, realism, and entertainment and not just more reading. “Playing with Tweezers” is 1 example of educational gaming as an innovative teaching strategy in nursing, as it encourages the strategy of involving the use of educational games to support the theoretical or practical classroom teaching. The teacher does not work with only 1 type of teaching methodology, but with a combination of methodologies. In addition, we cannot forget that skill training in educational games does not replace the curricular practice, but helps.

## Abbreviations

CPR: Cardiopulmonary Resuscitation
